# Measuring team science: Associations between a clinical-translational science institute and investigator ego networks

**DOI:** 10.1017/cts.2019.2

**Published:** 2019-05-14

**Authors:** Felichism W. Kabo, George A. Mashour

**Affiliations:** 1 Institute for Social Research, University of Michigan, Ann Arbor, MI, USA; 2 Michigan Institute for Clinical and Health Research, University of Michigan, Ann Arbor, MI, USA; 3 Office of Research, University of Michigan, Ann Arbor, MI, USA

**Keywords:** Ego networks, CTSA, network dynamics, collaboration, team science

## Abstract

The National Institutes of Health’s Clinical and Translational Science Awards (CTSA) institutes have been created, in part, to have a positive impact on collaboration and team science. This study is the first to examine the associations between a CTSA hub, the Michigan Institute for Clinical and Health Research (MICHR), and investigators’ ego networks. We ran cross-sectional and panel models of the associations between consulting with MICHR and the ego network measure of two-step reach (TSR) – that is, colleagues of colleagues reachable in two steps – from a network of 2161 investigators who had co-submitted a grant proposal to an external sponsor in 2006. Our analyses covered the period 2004–2012, although some model specifications covered the shorter time period 2006–2010. Consulting with MICHR had positive associations with the size of and changes in an investigator’s TSR across and over time, even controlling for research productivity and organizational affiliation. For example, over the period 2006–2010 an investigator who consulted with MICHR reached 44 more individuals than a non-consulting investigator. This study expands our understanding of the indirect impacts that clinical and translational science institutes have on investigators’ scientific networks. This network-based approach might be useful in quantifying the impact of team science initiatives at the university level.

## Introduction

The NIH Roadmap was developed to address the complexities of biomedical science and to accelerate scientific progress by tackling challenges that cut across NIH’s institutes and centers [1, 2]. The roadmap identified three major themes: (1) New Pathways to Discovery, (2) Research Teams of the Future, and (3) Re-engineering the Clinical Research Enterprise [1, 2]. The Clinical and Translational Science Awards (CTSA) program was launched in 2006, primarily to address the second and third of these themes [3]. However, the methods by which to assess the impact of a CTSA program hub on the development of research teams in clinical and translational science are still unclear.

CTSA program hubs were expected to catalyze clinical and translational research across the nation through activities such as training and cultivation of a translational science workforce, and the fostering of collaborative, interdisciplinary team science [4–6]. There is burgeoning evidence for the positive impact of CTSAs on a range of outcomes such as grant collaboration, publications, and citations [7–9]. The current study takes a novel approach in which, rather than focus on outcomes, we examine the antecedent issue of the potential means by which CTSAs are influencing the processes associated with the positive outcomes. We do this by identifying an individual-level mechanism through which a CTSA is transforming clinical and translational science. In particular, social network analysis is applied to advance understanding of how interactions with a CTSA program hub can influence the individual or ego networks of an investigator.

Social networks contribute to knowledge creation, which is a collective and social activity [10]. In this study we analyzed the impact of a CTSA program hub, the Michigan Institute for Clinical and Health Research (MICHR), on investigator ego networks at the University of Michigan (U-M) to assess the influence the institute has on the conditions that favor team science. MICHR is one of over 50 hubs of the CTSA program supported by the National Center for Advancing Translational Sciences (NCATS) of the National Institutes of Health (NIH). One of MICHR’s stated goals is to help enrich investigators’ research programs by connecting them to other units and individuals on campus. However, the most appropriate method by which to quantify enhanced scientific connectivity is unclear, both for MICHR and other CTSA hubs.

Previous network studies of CTSA program hubs have focused on changes of entire networks or communities of investigators. This type of approach is also referred to as *socio-centric* analysis. For example, network analysis was employed to assess collaboration, team science efforts, and inter-community cross-talk among researchers following the formation of a CTSA [7, 8, 11]. Another study focused on the design and implementation of a social network-based intervention to foster cross-disciplinary team science [12]. The current study differs from these previous investigations by instead focusing on the networks of *individuals*, which consist of the focal node (the individual or “ego”) and all of the other individuals to which the ego is directly connected (also called “alters”). This type of approach is referred to as *ego-centric* analysis. Generally, our study examines the impact of institutional interventions on individual ego networks. Specifically, we tested the hypothesis that consulting with MICHR would transform an investigator’s ego network by expanding the number of individuals that are two steps removed from the focal individual.

## Materials and Methods

### Data and Sample

**Fig. 1.**
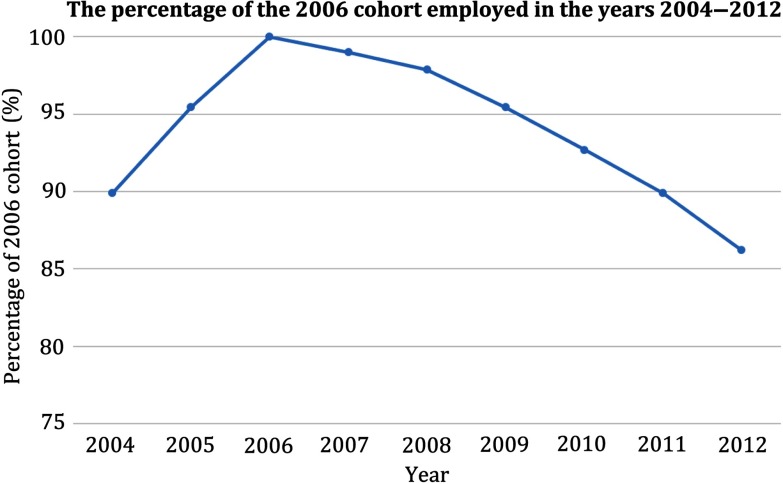
The percentage of the 2006 cohort that was employed at University of Michigan (U-M) for each of the years 2004–2012. Note that complete employment data were available for *N* = 1604 of the investigators in the 2006 cohort.

We studied a panel of 2161 investigators at U-M who submitted proposals to external sponsors in 2006. Our data allow us to track individuals on the basis of proposal submission activity, rather than on whether they were working at the university during that time period. While proposal submission activity is a better indicator of an investigator’s likelihood of contacting MICHR than is merely working at U-M, we nonetheless examined how many of the 2006 cohort were employed at U-M during the study period. We were able to do this for the 1604 investigators for whom we found complete human resource data. We found that the cohort attenuated as one moves forward or back in time with respect to 2006. For example, from Fig. 1, 90% of the cohort was employed at U-M in 2004, and 86% of the cohort was still employed at U-M in 2012.

Using the above sample, we ran cross-sectional and panel analysis of the links between MICHR and ego network reach. For the cross-sectional analyses, we focused on interactions with MICHR in 2006 (because this is the year that the institute was formally founded, followed by the CTSA award one year later in 2007) and on changes in ego network reach between 2004 and 2012. Note that we did robustness checks using models for consultation with MICHR in each of the years 2007–2010, and the results were almost identical. For the panel analysis, we focused on changes in ego network reach over the five-year period from 2006 to 2010. Table 1 shows the breakdown in the sample of 2161 investigators with respect to consulting with MICHR between 2006 and 2010.

**Table 1.** Yearly panel breakdown for consulting with MICHR, 2006–2010 (no new investigators added after 2006)

Year	Did not consult MICHR	Consulted MICHR	Total
2006	2033	128	2161
2007	2040	121	2161
2008	2007	154	2161
2009	1989	172	2161
2010	1934	227	2161

MICHR, Michigan Institute for Clinical and Health Research.

It is worth noting that there were low correlations between the subsamples that consulted with MICHR from year to year in the period 2006–2010. For example, 128 individuals consulted in 2006, 172 in 2009, and 227 in 2010 (Table 1). However, fewer individuals consulted from year to year: 42 consulted in both 2006 and 2009, 64 in both 2006 and 2010, and 93 in both 2009 and 2010. In other words, those consulting in one year may not necessarily consult in the following or other years. The findings imply that the associations between consulting with MICHR and changes in investigators’ ego networks cannot be explained as a result of repeated interactions with the exact same subsample of individuals.

We extracted administrative data, grant submissions to external sponsors, and publications data from the U-M Data Warehouse for the time period 2004–2012. We also retrieved administrative Tracking Metrics and Reporting System (TMRS) data from MICHR in order to identify which investigators had consulted with this CTSA in a given year. We therefore created our research dataset by merging U-M Data Warehouse and TMRS data.

### Variables

We used two-step reach (TSR) – that is, the number of all other individuals (colleagues, and colleagues of colleagues) in the network that are no more than two steps away from the focal individual – as our *dependent variable*. More specifically, we were interested in examining the changes in TSR between the time window before the investigator consulted with MICHR, and in the years following this consultation with MICHR. We did sensitivity analysis using other pre–post windows, for example, 2002 and 2012, and the results were similar. For the cross-sectional analysis, we created a variable, *ΔTwo-step Reach*, by calculating the difference in TSR between the pre-consultation and post-consultation years. We also examined whether the baseline TSR for a given window had any mediation on our models. For example, for the window between 2004 and 2012, we examined the association with TSR for 2004. Lastly, for the panel analysis, the yearly TSR was used as the dependent variable.

The *independent variable* was whether an investigator consulted with MICHR (the list of services offered by MICHR is available in Supplementary Table S1) in 2006. For robustness checks, we ran models that assessed the associations with multiple MICHR consultations for the years 2006–2010. The models were similar to those of the binary condition with respect to consultation. This suggests that whether or not an investigator consulted with MICHR was more important than the number of times the investigator consulted with MICHR (also see Supplementary Tables S2(a) and S2(b) and Figs. S1–S5; the figures show that, of those who consulted MICHR in any given year, the majority consulted with MICHR once; in fact, about 80% or more consulted MICHR three or fewer times). Therefore, we focused our analysis on whether or not an investigator consulted with MICHR as opposed to assessing multiple consultations. For the cross-sectional analysis we named this dichotomous variable *Consulted MICHR in 2006*, which was coded 1 if the investigator consulted with MICHR, and coded 0 otherwise. For the panel analysis, the yearly *Consulted MICHR* for the years 2006–2010 was the independent variable (also see Supplementary Table S3).

The *control variables* were quantitative (number of publications) and qualitative (mean journal impact factor) dimensions of scholarly output, organizational affiliation (*AFFILIATION*) at the college/school/unit level, gender (*GENDER*), and race (*RACE*). Control variables are critical to our understanding of the relationship between dependent and independent variables. They could impact the dependent variable even if we are not particularly interested in them. Therefore, in order to remove their confounding from our models, we held these variables constant or controlled for them, as failure to do so might invalidate any association we would find between the dependent and independent variables. For the cross-sectional analysis, the number of peer-reviewed publications in a given year was used as a proxy for the yearly quantitative dimensions of scholarship (*Number of publications in 20XX*, where XX are the last two digits of a specific year between 2004 and 2012). The mean journal impact factor of these publications was used as a proxy for the yearly qualitative dimensions of scholarship (*Mean Journal Impact Factor for pubs in 20XX*, where XX are the last two digits of a specific year between 2004 and 2012). Out of the 2161 individuals in the sample, we were able to collect organizational affiliation data for a subset of 1443 individuals (or 68.8% of the sample). Sensitivity analysis showed that there was no difference with respect to consultation with MICHR when we ran models with and without the affiliation variable. Publication data were only available for U-M units having subscription access to *Michigan Experts*, a proprietary service offered by Elsevier Publishing. *Michigan Experts* is a searchable database that contains publication citations for faculty affiliated with subscribing U-M units. Therefore, the models that controlled for productivity were restricted to the 1346 investigators affiliated with subscribing units, comprising 62.3% of the overall sample or 93.3% of the subsample that had organizational affiliation data. We conducted sensitivity analysis in which we ran productivity models without the affiliation variable, and following that without the publication variables. These models gave similar results to the fully specified models (and affiliation and controls for productivity) with respect to consultation with MICHR. Further, we found no differences in demographic characteristics between those who have publication and affiliation metrics, and those who do not. With respect to gender and racial differences, for example, investigators without publication and affiliation metrics were 26.8% female, 76.8% white, and 16.6% black, while those with publication and affiliation metrics were 26.8% female, 75.8% white, and 17.7% black. For the panel analysis the yearly *Number of publications* and *Mean Journal Impact Factor* for the years 2006–2010 were used to control for research productivity.

### Research Design

We utilized a historical cohort study design for the cross-sectional analysis of the change in ego network TSR between 2004 and 2012, and for the panel analysis of TSR for the period 2006–2010. Note that for the cross-sectional models, we further limited our analysis to the panel of investigators who submitted grants to external sponsors in 2006. This enabled us to compare the two groups with respect to the treatment or exposure factor of consultation or interaction with MICHR in 2006. Thus, the years 2004 and 2005 were considered pre-treatment, and the years 2007–2012 were considered post-treatment. For the panel analysis, we ran fixed-effects models with no time-invariant variables such as gender and race.

### Statistical Analysis

For the cross-sectional analysis we divided the study sample into two groups: *Consulting Group* was composed of investigators who interacted with MICHR in 2006, and *Non-Consulting Group* comprised investigators with no interaction with MICHR in the same year. We then ran ordinary least squares regression analysis specified as
(1)
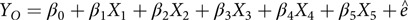

where

- *Y*_O_ → ΔTwo-step Reach,
- *X*_1_ → Number of publications in 20XX,
- *X*_2_ → Mean Journal Impact Factor for pubs in 20XX,
- *X*_3_ → *GENDER*,
- *X*_4_ → *RACE*,
- *X*_5_ → *AFFILIATION*.

We ran models for all pre- and post-years in the time period 2004–2012 both with and without the scholarship, demographic, and organizational controls in order to test the robustness of consulting with MICHR. We also ran models that adjusted for the baseline value of TSR in order to ascertain whether the baseline ego network may be a predictor of consulting with MICHR, rather than the other way around.

For the panel analysis we ran a Hausman test with the null hypothesis being that the preferred model reflects random effects, as opposed to the alternative fixed-effects specification, in order to decide between fixed or random-effects models [13]. We ran a fully specified fixed-effects model (with controls for gender, race, and affiliation) and saved the estimates, repeated the process for a random model, and then performed the Hausman test. The test results (χ^2^ = 133.23, *p* < 0.001) indicated that we should use fixed effects. Thus, we ran panel fixed-effects models with no time-invariant variables (such as gender or race) that are specified as
(2)
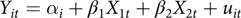

where

- *Y_it_* → *i* = ΔTwo-step Reach, *t* = *time*,
- *α_i_* → (*i* = 1, …, *n*) is the unknown intercept for each individual (*n* individual-specific intercepts),
- *X*_1_*_t_* → Number of publications, *t* = time,
- *X*_2_*_t_* → Mean Journal Impact Factor, *t* = time,
- *u_it_* → error term.

### Network Analysis

We generated university-wide networks using UCINET [14] software where a network tie or edge was constructed between a pair of investigators at U-M if they had co-submitted a grant proposal to an external sponsor in the years 2004–2012. We extracted ego networks for individuals in the study sample and generated TSR – the number of nodes that can be reached within two steps in a given ego network – as our proxy for information-related aspects of ego networks. TSR provides an assessment of the importance of “weak” ties (colleagues of colleagues) with respect to information and knowledge flows. This measure provides a richer view of the network resources available to an individual in comparison to the commonly used measure of degree centrality, equivalent to one-step reach.

**Fig. 2.**
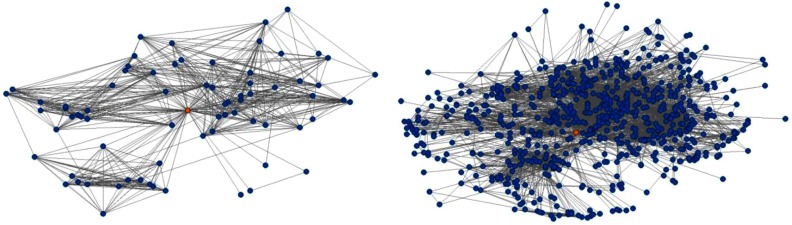
Differences between one-step reach (“degree,” 75 nodes or other individuals; left panel) and two-step reach (TSR), 686 nodes or other individuals; right panel) in terms of network size and structure. TSR captures the network resources directly and indirectly available to an individual, while degree only captures direct connections to others.

From Fig. 2 we see that, even for a very well-connected individual (the orange network node in the center), there is a substantial difference in the number of other nodes (individuals) in their degree (or one-step reach) and TSR neighborhoods. In the case shown in Fig. 2 the TSR neighborhood has over nine times as many nodes or individuals as the degree neighborhood.

## Results

### Cross-Sectional Analysis

**Fig. 3.**
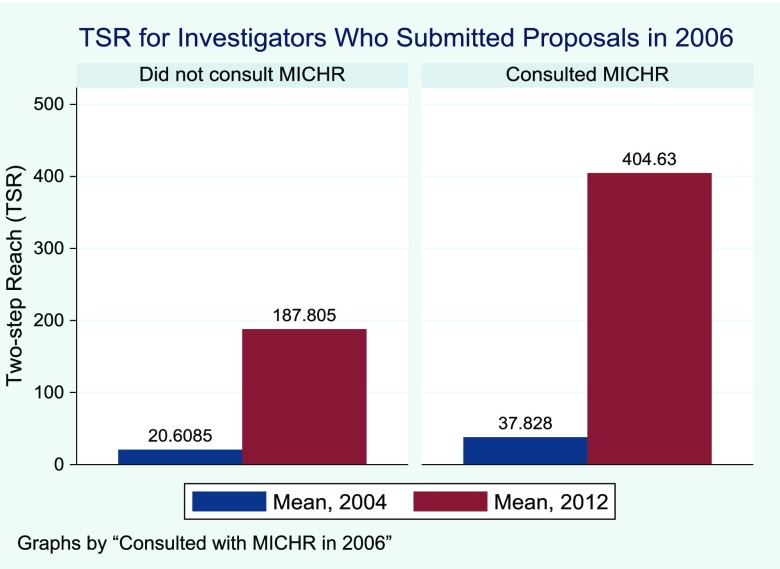
Two-step reach (TSR) scores for the 2161 investigators who submitted grant proposals to external sponsors in 2006. Scores are broken down by whether investigators consulted Michigan Institute for Clinical and Health Research (MICHR) in 2006 (*n* = 128) or not (*n* = 2033).

We analyzed the association between an investigator consulting MICHR in 2006 (treatment year) and ΔTSR in the individual’s ego network for a nine-year window in the years 2004–2012 (see Fig. 3 for a breakdown of TSR scores by MICHR consultation status, i.e., *Non-Consulting Group* versus *Consulting Group*).

**Fig. 4.**
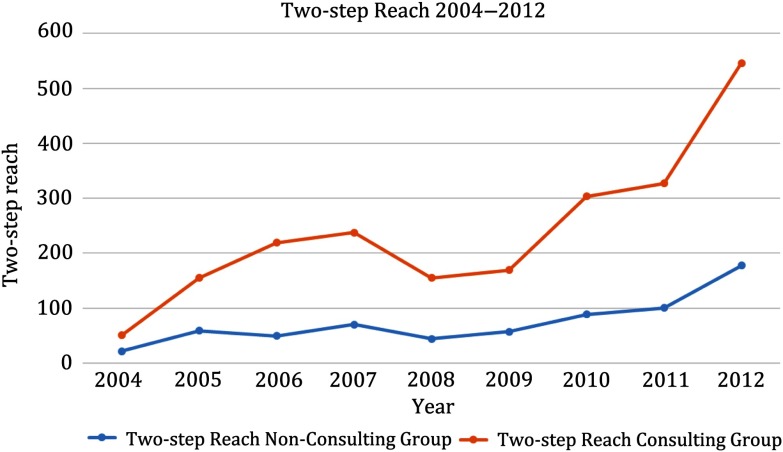
Comparison of yearly two-step reach (TSR) between *Consulting Group* and *Non-Consulting Group*. The two-step reach for *Consulting Group* increased faster than that for *Non-Consulting Group* even though the former had a higher baseline than the latter.

**Fig. 5.**
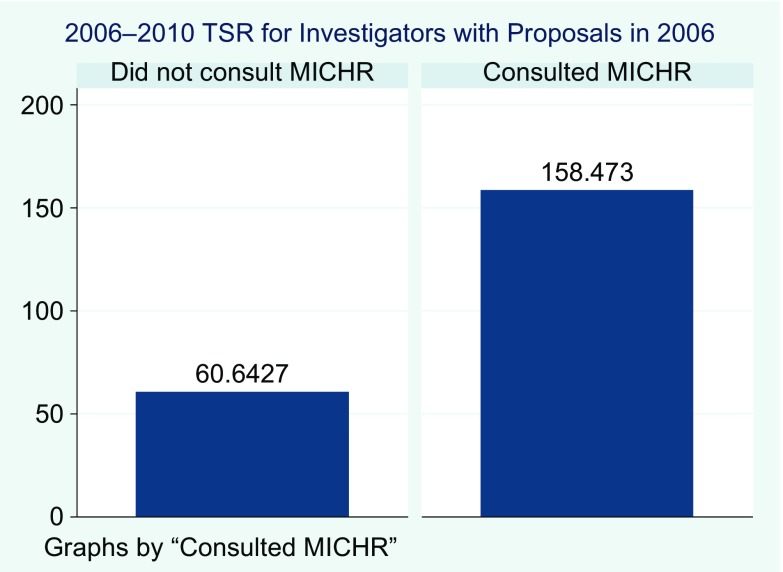
Two-step reach (TSR) values for the period 2006–2010 for investigators who submitted grant proposals to external sponsors in 2006. Scores are broken down by whether investigators consulted Michigan Institute for Clinical and Health Research (MICHR) or not.

We focused on the panel of investigators that submitted proposals to external sponsors in 2006. Therefore, we did not add any new investigators who submitted proposals to external sponsors after 2006. We also did not examine interactions between members of the panel and MICHR post-2006. We employed a pre–post design with respect to the treatment year (2006) and generated two sets of regression models as follows: (1) investigators from all units but with no controls for productivity (publications), and (2) investigators from subscribing (*Michigan Experts*) units, controlling for yearly quantitative (number of publications) and qualitative (mean journal impact factor of publications) research productivity in the time period corresponding to the years prior to and years subsequent to the treatment year. With respect to the second set of models, *Non-Consulting Group* had TSR scores in 2004 that were 0.54 time the size of *Consulting Group*’s (two-sample *t*-test: *t* = −5.85, *p* < 0.001). However, the difference between the two groups increased following 2006 (treatment year) such that in 2012 *Non-Consulting Group* had TSR scores that were 0.46 time the size of *Consulting Group*’s (two-sample *t*-test: *t* = −8.15, *p* < 0.001). In order to ascertain whether *Consulting Group* already had a two-fold greater TSR and continued along that trajectory regardless of consultation with MICHR, we examined the yearly TSR between the two groups (Fig. 4) and found that *Consulting Group* had a higher growth rate (also see Fig. 5 in the next subsection, “Panel Analysis”).

As noted earlier, the baseline TSR was greater for those who consulted with MICHR, even before MICHR existed in its current form as a CTSA program hub. Therefore, we ran models where we adjusted for baseline TSR as follows. For the 2004–2012 window, we included the TSR for 2004 in order to perform mediation analysis and to better understand the direction of associations in our model. Given the differences in baseline TSR between the *Non-Consulting Group* and the size of *Consulting Group*, a countervailing hypothesis to our theoretical framework would be one in which the baseline TSR predicts consulting with MICHR, as opposed to the other way around. We did the mediation analyses using 20 windows beginning (baselines) in 2002, 2003, 2004, or 2005, and ending in 2008, 2009, 2010, 2011, and 2012. For example, with 2002 as the baseline, the five windows were 2002–2008, 2002–2009, 2002–2010, 2002–2011, and 2002–2012. The evidence was mixed with respect to the association with baseline TSR. For most models with windows ending in 2008 and 2009, the baseline TSR was a suppressor (Supplementary Table S4). Conversely, for most models ending in 2011 and 2012, the baseline TSR was a mediator. For most models baselined in 2004, the baseline TSR was a mediator. Finally, for all models baselined in 2005, the baseline TSR was a suppressor. The analysis showed that the association with the baseline ego network was primarily a function of the study window selected for the 2006 cohort. Given the variation in the direction of the associations with baseline TSR values, we found an exhaustive discussion of the reasons behind this phenomenon to be beyond the scope of this paper. Further, consulting with MICHR was significant regardless of whether the baseline TSR mediated, suppressed, or did nothing. Therefore, we limited our analyses and subsequent discussion to the regression models without baseline TSR.

We present results for the two sets of models as follows: (1) for investigators from all units, we examined the relationship between consulting MICHR and TSR in each of the years 2006–2010 (Table 2), and (2) for investigators from subscribing (*Michigan Experts*) units, we analyzed the association between consulting with MICHR in 2006 and the change in ego network reach between 2004 and 2012 (Table 3). To economize on space, we show only the control variables (or levels within variables) that are significant (see Supplementary Tables S2a and S3 for the full models). We performed sensitivity analysis where we also generated models using different pre–post windows, for example, 2002 and 2012. The results were very similar to the ones we obtained using the window between 2004 and 2012. This implies that, despite different specifications of the pre–post window, there was no change in the way that consulting with MICHR impacted the dependent variable of Δ in *Two-step Reach* between 2004 and 2012.

**Table 2.** Cross-sectional models for the association with consulting MICHR in the years 2006–2010

	(1)	(2)	(3)	(4)	(5)
Year consulted MICHR	2006	2007	2008	2009	2010
**Variables**					
Consulted MICHR	160.9*** (30.52)	176.8*** (28.48)	92.15*** (27.12)	70.64** (25.51)	175.6*** (22.40)
**Affiliation**					
Medical school (reference category)					
College of engineering	−167.3*** (21.32)	−162.3*** (21.25)	−169.5*** (21.74)	−173.0*** (21.74)	−142.0*** (21.40)
Literature, science, arts	−156.4*** (31.35)	−151.5*** (31.20)	−159.6*** (31.80)	−162.8*** (31.86)	−140.0*** (30.92)
Life sciences institute	198.1^+^ (110.9)	208.1^+^ (110.3)	199.4^+^ (112.0)	196.6^+^ (112.2)	218.4* (109.0)
Constant	222.4*** (18.97)	226.2*** (18.60)	229.1*** (19.28)	233.7*** (19.19)	201.4*** (18.99)
Observations	936	936	936	936	936

MICHR, Michigan Institute for Clinical and Health Research.

Standard errors in parentheses. Dependent variable is Δ in *Two-step Reach* between 2004 and 2012 (all U-M investigators; no new investigators added after 2006).

***p < 0.001; **p < 0.01; *p < 0.05, ^+^*p* < 0.1.

**Table 3.** Cross-sectional models for the association with consulting MICHR in 2006

	(1)	(2)	(3)	(4)	(5)	(6)	(7)	(8)	(9)
Year of productivity	2004	2005	2006	2007	2008	2009	2010	2011	2012
**Variables**									
Consulted MICHR in 2006	162.3*** (30.62)	150.7*** (30.60)	152.7*** (30.33)	150.3*** (29.95)	155.6*** (30.15)	155.3*** (30.20)	149.3*** (30.54)	153.9*** (30.36)	147.6*** (30.32)
Number of publications	3.710** (1.139)	4.408*** (1.003)	5.505*** (0.983)	6.591*** (0.968)	5.398*** (0.944)	5.310*** (0.898)	4.412*** (0.904)	5.209*** (1.099)	7.633*** (1.394)
Mean JIF for pubs	2.491 (1.871)	1.187 (1.908)	0.834 (1.764)	2.400 (1.979)	3.593^+^ (1.863)	1.617 (1.572)	0.717 (1.507)	3.024^+^ (1.779)	2.904 (1.774)
**Affiliation**									
Medical school (reference category)									
College of engineering	−158.9*** (21.25)	−158.2*** (21.32)	−153 (21.08).0***	−148.1*** (21.00)	−148.0*** (21.12)	−150.2*** (21.05)	−151.3*** (21.28)	−155.7*** (21.07)	−169.3*** (21.40)
Literature, science, arts	−143.3*** (31.36)	−134.2*** (31.26)	−128.1*** (31.08)	−121.3*** (30.82)	−133.6*** (31.07)	−124.3*** (30.96)	−132.1*** (31.20)	−139.0*** (30.97)	−149.7*** (30.61)
School of public health	74.97* (33.24)	76.41* (33.07)	82.68* (32.91)	88.22** (32.52)	79.30* (32.68)	82.53* (32.74)	74.89* (32.96)	72.34* (32.82)	50.35 (32.70)
Constant	193.7*** (20.98)	190.0*** (20.71)	176.4*** (20.93)	158.8*** (21.01)	167.8*** (20.87)	168.8*** (20.93)	184.9*** (20.87)	176.2*** (21.01)	175.8*** (20.60)
Observations	873	873	873	873	873	873	873	873	873

JIF, journal impact factor; MICHR, Michigan Institute for Clinical and Health Research.

Standard errors in parentheses. Dependent variable is Δ in *Two-step Reach* between 2004 and 2012 (“*Michigan Experts*” investigators; no new investigators after 2006).

***p < 0.001, **p < 0.01, *p < 0.05, ^+^*p* < 0.1.

### Panel Analysis

We present models with (model 2, Table 4) and without (model 1, Table 4) controls for research productivity. Recall that model 1 captures all investigators in the panel, while model 2 only captures investigators in the panel who are affiliated with “*Michigan Experts*” units. The panel regressions revealed that consulting with MICHR had a robust, significant, and positive longitudinal association with ego network TSR for the period 2006–2010.

**Table 4.** Panel model for the association between consulting with MICHR and two-step reach between 2006 and 2010 (no new investigators added after 2006)

	(1)	(2)
	All investigators	“Michigan Experts” only
Variables	Two-step reach	Two-step reach
Consulted MICHR	44.16*** (3.829)	43.88*** (4.403)
Number of publications		0.346 (0.256)
Mean journal impact factor for pubs		−0.0546 (0.261)
Constant	65.84*** (0.900)	67.73*** (2.554)
Observations	8280	5914
Number of individuals	2161	1346

MICHR, Michigan Institute for Clinical and Health Research.

Standard errors in parentheses.

***p < 0.001.

The findings for the models with and without controls for research productivity are similar. Therefore, we focused our analysis on model 1 as it allows us to discuss the association with consulting MICHR for the entire panel. Over time (2006–2010), the act of an investigator consulting with MICHR was associated with an increase in TSR by roughly 44 units (44.16 for the model without publications, and 43.88 for the model with publications) – that is, the investigator was able to reach 44 more individuals in two steps compared with a peer who did not consult with MICHR.

## Discussion

### Cross-Sectional Analysis

We employed cross-sectional analysis to examine the association between consulting with MICHR in each of the years 2006–2010 and changes in TSR across the nine-year window between 2004 and 2012. We found that consulting with MICHR had a positive and significant association with Δ in TSR between 2004 and 2012. This association was large and robust to differences when, during the 2006–2010 window, an investigator consulted with MICHR. We then focused on the association with consulting with MICHR in 2006. Our analysis showed that, even with controls for research productivity for each of the years in the span 2004–2012, there was a positive and significant association between consulting with MICHR and Δ in TSR for the period between 2004 and 2012.

### Panel Analysis

Following the cross-sectional analysis, we ran panel regressions in order to ascertain the temporal associations with consulting with MICHR. The panel models revealed a positive and significant longitudinal association between consulting with MICHR and TSR for the time period 2006–2010. Controlling for research productivity had little impact on the size of the association between consulting with MICHR and TSR.

### Synthesis and Implications

Both sets of analyses (cross-sectional and panel) revealed that consulting with MICHR had large, positive, and significant associations with the structure of an investigator’s ego network. The cross-sectional analysis enabled us to examine the network associations with MICHR on a year-to-year basis, while the panel analysis allowed us to probe these network associations over time. The cross-sectional analysis provided insights into the associations with MICHR on a population of U-M investigators at specific points in time, as we were able to use multiple variables to assess the association between MICHR and change in ego networks regardless of whether the variable varied over time. The panel analysis employed fewer variables (no time-invariant variables), but allowed tracking of changes over time in the association between consulting MICHR and an investigator’s ego network. The two types of analysis have complementary strengths and weaknesses. Thus, simultaneously employing both types of analysis enabled us to demonstrate the robustness of the association between consulting MICHR and changes in an investigator’s ego network.

Our review of the literature established that there has been little or no research on how institutions transform ego networks in the context of knowledge creation or transformation. Most studies of the dynamics of ego networks have focused on the links between a set of behaviors and structural network changes. For example, a study found that the number of prosocial activities that a person engages in is positively associated with an increase in network degree or having more friends [15]. Another study of individuals entering a new institutional setting found that core discussion ego networks change rapidly as a consequence of obligations and routine activities being transformed by the new institutional environment [16].

Of closer relevance to our work, a study of changes in ego networks of knowledge workers over time identified potential causal mechanisms behind temporal network changes [10]. The authors demonstrated that ego network changes are spurred by the need for the types of knowledge resources that are embodied in potential interaction partners [10]. While the authors discuss potential interaction partners primarily in terms of individuals, we argue that CTSA program hubs, such as MICHR, can also fill that role.

There are three potential pathways by which a CTSA could positively impact an individual’s ego network cross-sectionally and longitudinally. First, the CTSA can directly enhance the individual’s intellectual capital by, for example, making the person more knowledgeable about the grant writing process and thus making the individual a more attractive partner for others. Further, given that we have previously established that a CTSA can have significant impacts on whether an investigator receives a grant award and on the size of the award [17], the receipt of grant could also enhance the investigator’s status and their desirability as an interaction partner. Second, the CTSA can boost an individual’s social capital by, for example, facilitating mentorship by more experienced investigators. This impacts knowledge creation indirectly by enriching the individual’s ego network. The focal individual would also presumably benefit from her or his mentor’s network contacts, thus obtaining opportunities to increase ego network reach over time. Our working hypothesis was that interacting with MICHR would likely result in significant benefits to the focal individual in the form of a richer ego network or an expanded ego network reach. The greater an individual’s ego network reach, the better connected he or she is to potential network resources, including advice and research collaborations. The results from this study support the hypothesis that consulting with MICHR is positively and significantly associated with the structure of investigators’ ego networks. Third, there can be benefit from the interdisciplinary nature of the CTSA itself. The CTSAs are designed to “break down silos” through mechanisms such as introducing investigators to others who have common interests but who are outside of their academic departments. This match-making process has the potential to catalyze collaboration across departmental boundaries, but it could also lead to qualitative and quantitative changes in an investigator’s ego network.

### Limitations

There are limitations to this study, especially in relation to scope and methodology. The first limitation is that we did not have any means of identifying the pathways and career trajectories that investigators followed in the years preceding the study. The ability to record these pathways and trajectories would have enhanced our capacity to explain phenomena such as why some investigators had different baseline values with respect to the sizes of their ego networks. For example, Fig. 3 shows that in 2004 the investigators who consulted MICHR (mean = 37.83) in 2006 had TSR scores that were larger than for investigators that did not consult MICHR (mean = 20.61). By 2012 the difference in TSR scores between the two groups (*Consulting Group* mean = 404.63, *Non-Consulting Group* mean = 187.80) had increased significantly (Fig. 4). On the surface it would appear that MICHR may contribute to the “Matthew Effect” [18], where those with better ego network structures benefit even more. Alternatively, it could be that specific pathways or trajectories condition investigators to be more or less likely to consult with MICHR. However, we did not have the data to examine these issues and control for these potential confounds. The fact that we could not examine investigators’ prebaseline trajectories constrained our ability to extrapolate to causation, while doing so would have amplified the policy relevance of our work for MICHR and other CTSAs. Nonetheless, it is worth recalling that, despite our inability to capture pre-baseline trajectories, consulting with MICHR was associated with higher TSR growth rates following the baseline, suggesting the association was robust to the limitation.

The second limitation is related to the fact that we used fixed-effects panel regressions to analyze the impact of consulting with MICHR over time. In using the fixed-effects models we assumed that something intrinsic to the investigators may bias either our dependent (TSR) or independent (consulting with MICHR) variables, hence the need to control for this. The utility of the fixed-effects model is that it removes the association with time-invariant factors such as gender or race so that we can assess the net association between consulting with MICHR (and research productivity) and an investigator’s TSR. However, this also means that we cannot use the fixed-effects model to examine the links between time-invariant factors such as gender, race, and (arguably) affiliation and our dependent variable (TSR).

The third limitation is that the study was conducted at only one CTSA program hub, which has implications on the generalizability of our findings to other CTSAs and even non-CTSA institutes. Although it might be reasonable to assume that our findings should hold for U-M peer institutions (i.e., large research universities) that have CTSA program hubs, more robust claims are only possible with replication studies at other CTSAs.

## Conclusion

We demonstrated that MICHR, a CTSA program hub, has significant associations with the structure of and changes in the ego networks of individual investigators. This is a crucial step in expanding our understanding of the indirect impacts that institutes such as MICHR have on clinical and translational knowledge creation. Specifically, we found that consulting or interacting with MICHR was significantly correlated with aspects of network structure and with changes over time in investigators’ ego networks. Future studies in translational and team science could be applied to other or multiple CTSA hubs by building on this investigation to directly and simultaneously link the dimensions (such as TSR) and the dynamics of network structure (such as ΔTSR) to important outcomes such as successful awards, publications, or patents.

## Supplementary material

For supplementary material accompanying this paper visit https://doi.org/10.1017/cts.2019.2. click here to view supplementary material
