# Supplementary material for: Measuring team science: Associations between a clinical-translational science institute and investigator ego networks
Source: J Clin Transl Sci. 2019 May 14;2(6):363–70. doi: 10.1017/cts.2019.2 (PMC6676646; doi:10.1017/cts.2019.2)
Supplement: Supplementary file 1 [file S2059866119000025sup001.docx]

**Supplementary Material (Felichism W. Kabo, George A. Mashour)**

**Supplementary Table S1**. Services provided by MICHR. The TRMS records indicated that there were 23 different services that MICHR provided to investigators as shown in Supplementary Table S1 below. We were interested in whether an investigator made use of any of these services over the lifecycle of a grant proposal.

| **SERVICE NAME** |
| --- |
| Admin shell |
| Budget |
| Clinical Research Billing Calendar |
| Consult |
| Content development |
| Contract |
| Data Management |
| Feasibility Assessment |
| Find collaborator |
| Funding |
| Grant resubmit RDC 1st attempt |
| Grant resubmit RDC 2nd or subsequent attempt |
| Grant submission 1st attempt |
| IND/IDE submission |
| Informed Consent |
| IRB submission |
| Letter of Support |
| MCRU |
| Navigation |
| Other |
| Project Mgmt & Monitoring |
| Publication review |
| Registries |

**Supplementary Table S2a**. Full cross-sectional models for the association between consulting MICHR in each of the years 2006-2010 and two-step reach (all U-M investigators; no new investigators added after 2006).

|  | (1) | (2) | (3) | (4) | (5) |
| --- | --- | --- | --- | --- | --- |
| *Year Consulted MICHR* | 2006 | 2007 | 2008 | 2009 | 2010 |
| DV = ∆ Two-step Reach 2004 to 2012 |  |  |  |  |  |
| **VARIABLES** |  |  |  |  |  |
|  |  |  |  |  |  |
| Consulted MICHR | 160.9*** | 176.8*** | 92.15*** | 70.64** | 175.6*** |
|  | (30.52) | (28.48) | (27.12) | (25.51) | (22.40) |
| GENDER |  |  |  |  |  |
| Female |  |  |  |  |  |
| (reference category) |  |  |  |  |  |
| Male | 9.186 | -0.361 | 4.791 | 3.401 | 2.904 |
|  | (19.23) | (19.11) | (19.38) | (19.42) | (18.87) |
| RACE |  |  |  |  |  |
| White |  |  |  |  |  |
| (reference category) |  |  |  |  |  |
| Asian | -25.94 | -24.67 | -23.66 | -27.28 | -14.06 |
|  | (23.11) | (22.98) | (23.34) | (23.36) | (22.77) |
| Hispanic | 87.54+ | 69.16 | 87.34+ | 93.14* | 90.43* |
|  | (45.27) | (45.20) | (45.73) | (45.75) | (44.44) |
| Black | -26.89 | -10.70 | -17.73 | -16.55 | -12.19 |
|  | (48.78) | (48.50) | (49.19) | (49.31) | (47.91) |
| 2 or more | -137.6 | -137.6 | -140.8 | -143.2 | -122.9 |
|  | (109.2) | (108.6) | (110.2) | (110.4) | (107.3) |
| Not Indicated | -59.04 | -108.5 | -90.28 | -96.62 | -42.67 |
|  | (93.93) | (93.50) | (94.82) | (95.24) | (92.32) |
| Native American | -98.89 | -89.64 | -100.9 | -99.67 | -77.54 |
|  | (124.3) | (123.6) | (125.4) | (125.7) | (122.2) |
| Hawaiian | -190.6 | -153.4 | -186.5 | -186.2 | -153.7 |
|  | (262.6) | (261.2) | (265.0) | (265.5) | (258.0) |
| AFFILIATION |  |  |  |  |  |
| Medical School |  |  |  |  |  |
| (reference category) |  |  |  |  |  |
| College of Engineering | -167.3*** | -162.3*** | -169.5*** | -173.0*** | -142.0*** |
|  | (21.32) | (21.25) | (21.74) | (21.74) | (21.40) |
| Literature, Science, Arts | -156.4*** | -151.5*** | -159.6*** | -162.8*** | -140.0*** |
|  | (31.35) | (31.20) | (31.80) | (31.86) | (30.92) |
| School of Public Health | 62.62+ | 58.36+ | 60.02+ | 51.85 | 61.55+ |
|  | (33.42) | (33.16) | (33.77) | (33.70) | (32.77) |
| Benefits Pool & Reser | -85.53+ | -87.59+ | -83.55+ | -86.91+ | -61.21 |
|  | (45.84) | (45.53) | (46.43) | (46.52) | (45.22) |
| School of Dentistry | -70.22 | -80.64 | -63.08 | -67.16 | -48.80 |
|  | (53.29) | (53.02) | (53.79) | (53.88) | (52.42) |
| College of Pharmacy | -21.22 | -15.60 | -32.08 | -28.06 | -25.41 |
|  | (71.17) | (70.79) | (71.75) | (72.02) | (69.85) |
| School of Nursing | 19.38 | -26.28 | 1.210 | -10.29 | -25.77 |
|  | (87.99) | (87.41) | (88.65) | (88.83) | (86.34) |
| Life Sciences Institute | 198.1+ | 208.1+ | 199.4+ | 196.6+ | 218.4* |
|  | (110.9) | (110.3) | (112.0) | (112.2) | (109.0) |
| UMTRI | -149.2+ | -145.6+ | -152.5+ | -156.1+ | -123.3 |
|  | (81.90) | (81.44) | (82.69) | (82.86) | (80.59) |
| Institute for Social Research | -81.30 | -78.21 | -85.11 | -88.55 | -56.39 |
|  | (100.3) | (99.71) | (101.2) | (101.4) | (98.61) |
| Provost & EVPA | -77.84 | -99.21 | -84.14 | -95.94 | -115.3 |
|  | (83.24) | (82.65) | (84.00) | (84.06) | (81.68) |
| School of Kinesiology | -90.41 | -118.8 | -93.82 | -109.5 | -97.97 |
|  | (101.9) | (101.3) | (102.9) | (103.0) | (100.1) |
| School of Social Work | -12.05 | -43.41 | -18.43 | -21.97 | -21.54 |
|  | (100.2) | (99.52) | (101.1) | (101.3) | (98.34) |
| U-M Dearborn College of Engineering | -201.6 | -201.8 | -208.3 | -208.7 | -189.2 |
|  | (173.3) | (172.3) | (174.8) | (175.2) | (170.2) |
| School of Information | -348.6 | -342.8 | -350.9 | -354.1 | -321.3 |
|  | (243.2) | (241.8) | (245.4) | (245.9) | (239.0) |
| CHGD | 256.6 | 252.8 | 249.9 | 245.3 | 189.8 |
|  | (172.8) | (171.8) | (174.3) | (174.7) | (169.8) |
| President's Office | -83.55 | -83.18 | -89.17 | -91.30 | -65.28 |
|  | (172.4) | (171.4) | (173.9) | (174.3) | (169.4) |
| Vice President for Research | -23.73 | 66.91 | 52.33 | 45.49 | -96.15 |
|  | (173.7) | (172.5) | (175.1) | (175.4) | (171.1) |
| Mary Rackham Institute | -80.43 | -261.0 | -87.10 | -91.74 | -59.35 |
|  | (243.6) | (243.5) | (245.8) | (246.3) | (239.3) |
| TCAUP | -226.6 | -220.8 | -228.9 | -232.1 | -199.3 |
|  | (243.2) | (241.8) | (245.4) | (245.9) | (239.0) |
| Rackham Graduate School | -189.4 | -193.2 | -196.1 | -200.7 | -168.4 |
|  | (243.6) | (242.2) | (245.8) | (246.3) | (239.3) |
| SNRE | -216.6 | -210.8 | -218.9 | -222.1 | -189.3 |
|  | (243.2) | (241.8) | (245.4) | (245.9) | (239.0) |
| Constant | 222.4*** | 226.2*** | 229.1*** | 233.7*** | 201.4*** |
|  | (18.97) | (18.60) | (19.28) | (19.19) | (18.99) |
|  |  |  |  |  |  |
| Observations | 936 | 936 | 936 | 936 | 936 |
|  |  |  |  |  |  |
| Standard errors in parentheses |  |  |  |  |  |
| *** p<0.001, ** p<0.01, * p<0.05, + p<0.1 |  |  |  |  |  |

**Supplementary Table S2b**. Full cross-sectional models for the association between number of times an investigator consulted MICHR in each of the years 2006-2010 and two-step reach (all U-M investigators; no new investigators added after 2006).

|  | (1) | (2) | (3) | (4) | (5) |
| --- | --- | --- | --- | --- | --- |
| *Year Consulted MICHR* | 2006 | 2007 | 2008 | 2009 | 2010 |
| DV = ∆ Two-step Reach 2004 to 2012 |  |  |  |  |  |
| **VARIABLES** |  |  |  |  |  |
|  |  |  |  |  |  |
| Number of Times Consulted MICHR | 11.80** | 8.976+ | 17.78*** | 14.77** | 18.45*** |
|  | (4.399) | (5.054) | (3.547) | (5.468) | (4.206) |
| GENDER |  |  |  |  |  |
| Female |  |  |  |  |  |
| (reference category) |  |  |  |  |  |
| Male | 4.617 | 2.606 | 2.740 | 2.407 | 3.086 |
|  | (19.42) | (19.50) | (19.24) | (19.44) | (19.30) |
| RACE |  |  |  |  |  |
| White |  |  |  |  |  |
| (reference category) |  |  |  |  |  |
| Asian | -27.97 | -28.18 | -29.04 | -28.34 | -27.37 |
|  | (23.37) | (23.42) | (23.14) | (23.37) | (23.21) |
| Hispanic | 86.56+ | 96.28* | 80.83+ | 80.20+ | 82.97+ |
|  | (45.92) | (45.84) | (45.41) | (46.18) | (45.56) |
| Black | -20.21 | -17.99 | -15.96 | -19.47 | -15.34 |
|  | (49.31) | (49.43) | (48.84) | (49.31) | (48.99) |
| 2 or more | -148.2 | -149.6 | -146.5 | -147.7 | -144.2 |
|  | (110.4) | (110.7) | (109.4) | (110.4) | (109.7) |
| Not Indicated | -71.54 | -75.16 | -104.5 | -77.26 | -66.31 |
|  | (94.95) | (95.15) | (94.20) | (94.94) | (94.34) |
| Native American | -107.2 | -107.7 | -105.4 | -104.4 | -101.1 |
|  | (125.7) | (126.0) | (124.5) | (125.7) | (124.9) |
| Hawaiian | -187.5 | -184.7 | -186.0 | -186.3 | -179.7 |
|  | (265.6) | (266.2) | (263.0) | (265.6) | (263.8) |
| AFFILIATION |  |  |  |  |  |
| Medical School |  |  |  |  |  |
| (reference category) |  |  |  |  |  |
| College of Engineering | -179.8*** | -181.1*** | -175.6*** | -177.6*** | -172.4*** |
|  | (21.38) | (21.47) | (21.16) | (21.48) | (21.35) |
| Literature, Science, Arts | -170.8*** | -172.6*** | -166.2*** | -168.4*** | -168.1*** |
|  | (31.54) | (31.62) | (31.23) | (31.61) | (31.32) |
| School of Public Health | 54.39 | 52.10 | 48.47 | 53.13 | 56.89+ |
|  | (33.76) | (33.82) | (33.37) | (33.73) | (33.52) |
| Benefits Pool & Reser | -97.80* | -97.00* | -90.58* | -92.50* | -90.37* |
|  | (46.27) | (46.41) | (45.86) | (46.36) | (46.02) |
| School of Dentistry | -64.59 | -67.22 | -73.40 | -63.57 | -60.93 |
|  | (53.92) | (54.02) | (53.37) | (53.93) | (53.57) |
| College of Pharmacy | -35.53 | -37.71 | -32.61 | -33.00 | -32.55 |
|  | (71.90) | (72.07) | (71.19) | (71.94) | (71.42) |
| School of Nursing | 1.128 | -5.390 | 1.890 | -3.481 | -3.888 |
|  | (88.88) | (89.04) | (87.99) | (88.84) | (88.26) |
| Life Sciences Institute | 184.1 | 182.5 | 193.7+ | 186.2+ | 189.7+ |
|  | (112.1) | (112.4) | (111.0) | (112.1) | (111.4) |
| UMTRI | -164.1* | -165.6* | -160.3+ | -162.2+ | -156.8+ |
|  | (82.76) | (82.95) | (81.95) | (82.77) | (82.24) |
| Institute for Social Research | -96.22 | -97.64 | -92.78 | -94.00 | -89.43 |
|  | (101.4) | (101.6) | (100.4) | (101.4) | (100.7) |
| Provost & EVPA | -94.88 | -99.11 | -92.57 | -94.91 | -97.44 |
|  | (84.09) | (84.25) | (83.26) | (84.08) | (83.51) |
| School of Kinesiology | -104.3 | -107.5 | -101.1 | -110.6 | -107.5 |
|  | (103.0) | (103.2) | (102.0) | (103.0) | (102.3) |
| School of Social Work | -28.91 | -32.80 | -26.24 | -27.74 | -29.14 |
|  | (101.2) | (101.4) | (100.2) | (101.2) | (100.6) |
| U-M Dearborn College of Engineering | -215.6 | -217.5 | -211.4 | -214.0 | -209.4 |
|  | (175.2) | (175.6) | (173.5) | (175.2) | (174.0) |
| School of Information | -362.4 | -363.5 | -358.3 | -360.1 | -354.8 |
|  | (245.9) | (246.5) | (243.5) | (245.9) | (244.3) |
| CHGD | 238.2 | 235.1 | 240.5 | 238.3 | 235.0 |
|  | (174.7) | (175.0) | (173.0) | (174.7) | (173.5) |
| President's Office | -98.63 | -100.6 | -94.88 | -97.20 | -92.09 |
|  | (174.3) | (174.7) | (172.6) | (174.3) | (173.1) |
| Vice President for Research | 29.31 | 34.20 | 47.18 | 45.53 | 22.06 |
|  | (175.4) | (175.8) | (173.7) | (175.5) | (174.2) |
| Mary Rackham Institute | -98.80 | -110.8 | -96.53 | -98.67 | -92.73 |
|  | (246.3) | (246.9) | (243.9) | (246.3) | (244.7) |
| TCAUP | -240.4 | -241.5 | -236.3 | -238.1 | -232.8 |
|  | (245.9) | (246.5) | (243.5) | (245.9) | (244.3) |
| Rackham Graduate School | -207.8 | -210.9 | -205.5 | -207.7 | -201.7 |
|  | (246.3) | (246.8) | (243.9) | (246.3) | (244.7) |
| SNRE | -230.4 | -231.5 | -226.3 | -228.1 | -222.8 |
|  | (245.9) | (246.5) | (243.5) | (245.9) | (244.3) |
| Constant | 240.8*** | 243.9*** | 238.5*** | 240.7*** | 234.7*** |
|  | (18.74) | (18.72) | (18.51) | (18.75) | (18.69) |
|  |  |  |  |  |  |
| Observations | 936 | 936 | 936 | 936 | 936 |
|  |  |  |  |  |  |
| Standard errors in parentheses |  |  |  |  |  |
| *** p<0.001, ** p<0.01, * p<0.05, + p<0.1 |  |  |  |  |  |

**Supplementary Table S3**. Full cross-sectional models for the association between consulting MICHR in 2006 and ∆ in two-step reach between 2004 and 2012 (“*Michigan Experts*” investigators; no new investigators after 2006).

|  | (1) | (2) | (3) | (4) | (5) | (6) | (7) | (8) | (9) |
| --- | --- | --- | --- | --- | --- | --- | --- | --- | --- |
| *Year of Research Productivity* | 2004 | 2005 | 2006 | 2007 | 2008 | 2009 | 2010 | 2011 | 2012 |
| DV = ∆ Two-step Reach 2004 to 2012 |  |  |  |  |  |  |  |  |  |
| VARIABLES |  |  |  |  |  |  |  |  |  |
|  |  |  |  |  |  |  |  |  |  |
| Consulted MICHR in 2006 | 162.3*** | 150.7*** | 152.7*** | 150.3*** | 155.6*** | 155.3*** | 149.3*** | 153.9*** | 147.6*** |
|  | (30.62) | (30.60) | (30.33) | (29.95) | (30.15) | (30.20) | (30.54) | (30.36) | (30.32) |
| Number of publications | 3.710** | 4.408*** | 5.505*** | 6.591*** | 5.398*** | 5.310*** | 4.412*** | 5.209*** | 7.633*** |
|  | (1.139) | (1.003) | (0.983) | (0.968) | (0.944) | (0.898) | (0.904) | (1.099) | (1.394) |
| Mean Journal Impact Factor for pubs | 2.491 | 1.187 | 0.834 | 2.400 | 3.593+ | 1.617 | 0.717 | 3.024+ | 2.904 |
|  | (1.871) | (1.908) | (1.764) | (1.979) | (1.863) | (1.572) | (1.507) | (1.779) | (1.774) |
| GENDER |  |  |  |  |  |  |  |  |  |
| Female |  |  |  |  |  |  |  |  |  |
| (reference category) |  |  |  |  |  |  |  |  |  |
| Male | -5.817 | -11.01 | -9.232 | -7.636 | -11.36 | -7.090 | -8.440 | -6.016 | -7.174 |
|  | (19.77) | (19.78) | (19.52) | (19.27) | (19.42) | (19.41) | (19.62) | (19.49) | (19.40) |
| RACE |  |  |  |  |  |  |  |  |  |
| White |  |  |  |  |  |  |  |  |  |
| (reference category) |  |  |  |  |  |  |  |  |  |
| Asian | -27.46 | -31.44 | -39.44+ | -39.68+ | -34.33 | -35.54 | -32.63 | -37.55 | -39.71+ |
|  | (23.31) | (23.24) | (23.17) | (22.88) | (22.97) | (23.02) | (23.19) | (23.18) | (23.08) |
| Hispanic | 89.08+ | 87.29+ | 89.39+ | 71.46 | 80.59+ | 76.70+ | 77.79+ | 78.74+ | 67.05 |
|  | (46.06) | (45.89) | (45.56) | (45.17) | (45.40) | (45.43) | (45.85) | (45.70) | (45.59) |
| Black | -45.43 | -50.02 | -38.58 | -50.42 | -39.56 | -31.94 | -46.09 | -34.26 | -39.48 |
|  | (52.32) | (52.10) | (51.78) | (51.19) | (51.50) | (51.60) | (52.69) | (51.93) | (51.60) |
| 2 or more | -124.1 | -113.5 | -125.0 | -124.3 | -117.8 | -107.4 | -129.8 | -115.6 | -130.7 |
|  | (108.2) | (107.8) | (107.0) | (105.7) | (106.4) | (106.6) | (107.4) | (107.2) | (106.6) |
| Not Indicated | -64.97 | -48.58 | -52.61 | -55.53 | -73.33 | -51.11 | -41.00 | -50.94 | -65.62 |
|  | (92.97) | (92.62) | (92.03) | (90.89) | (91.59) | (91.59) | (92.43) | (92.11) | (91.73) |
| Native American | -147.3 | -145.6 | -130.8 | -127.2 | -95.01 | -117.4 | -92.68 | -99.80 | -103.6 |
|  | (123.7) | (122.9) | (121.9) | (120.3) | (121.1) | (121.2) | (122.4) | (121.9) | (121.3) |
| AFFILIATION |  |  |  |  |  |  |  |  |  |
| Medical School |  |  |  |  |  |  |  |  |  |
| (reference category) |  |  |  |  |  |  |  |  |  |
| College of Engineering | -158.9*** | -158.2*** | -153.0*** | -148.1*** | -148.0*** | -150.2*** | -151.3*** | -155.7*** | -169.3*** |
|  | (21.25) | (21.32) | (21.08) | (21.00) | (21.12) | (21.05) | (21.28) | (21.07) | (21.40) |
| Literature, Science, Arts | -143.3*** | -134.2*** | -128.1*** | -121.3*** | -133.6*** | -124.3*** | -132.1*** | -139.0*** | -149.7*** |
|  | (31.36) | (31.26) | (31.08) | (30.82) | (31.07) | (30.96) | (31.20) | (30.97) | (30.61) |
| School of Public Health | 74.97* | 76.41* | 82.68* | 88.22** | 79.30* | 82.53* | 74.89* | 72.34* | 50.35 |
|  | (33.24) | (33.07) | (32.91) | (32.52) | (32.68) | (32.74) | (32.96) | (32.82) | (32.70) |
| School of Dentistry | -43.60 | -47.82 | -36.87 | -31.89 | -32.25 | -36.06 | -52.62 | -57.28 | -57.09 |
|  | (53.25) | (52.78) | (52.52) | (51.84) | (52.27) | (52.25) | (52.54) | (52.32) | (52.11) |
| College of Pharmacy | -4.114 | 2.529 | 5.127 | 18.81 | 10.81 | -0.889 | 7.215 | -0.785 | -9.284 |
|  | (70.58) | (70.34) | (69.83) | (69.06) | (69.53) | (69.58) | (70.21) | (69.88) | (69.52) |
| School of Nursing | 30.90 | 32.70 | 29.29 | 35.56 | 35.94 | 50.68 | 30.59 | 36.10 | 22.22 |
|  | (87.25) | (86.90) | (86.25) | (85.28) | (85.82) | (86.03) | (86.66) | (86.42) | (85.97) |
| Life Sciences Institute | 191.5+ | 183.3+ | 192.7+ | 161.9 | 133.0 | 134.8 | 152.9 | 142.7 | 140.4 |
|  | (109.8) | (109.4) | (109.1) | (107.6) | (108.8) | (108.6) | (109.4) | (109.2) | (109.1) |
| UMTRI | -119.8 | -115.4 | -111.3 | -92.43 | -100.1 | -103.7 | -111.7 | -115.2 | -117.8 |
|  | (81.41) | (81.08) | (80.48) | (79.62) | (80.11) | (80.17) | (80.89) | (80.55) | (80.07) |
| School of Kinesiology | -60.46 | -63.05 | -52.31 | -54.03 | -45.18 | -71.28 | -77.08 | -81.35 | -94.14 |
|  | (101.3) | (100.7) | (99.99) | (98.71) | (99.49) | (99.40) | (100.3) | (99.96) | (99.45) |
| U-M Dearborn College of Engineering | -186.1 | -182.2 | -154.9 | -171.8 | -182.3 | -183.3 | -179.4 | -206.5 | -178.8 |
|  | (171.6) | (170.9) | (169.9) | (167.8) | (168.9) | (169.0) | (170.5) | (170.1) | (169.3) |
| Constant | 193.7*** | 190.0*** | 176.4*** | 158.8*** | 167.8*** | 168.8*** | 184.9*** | 176.2*** | 175.8*** |
|  | (20.98) | (20.71) | (20.93) | (21.01) | (20.87) | (20.93) | (20.87) | (21.01) | (20.60) |
|  |  |  |  |  |  |  |  |  |  |
| Observations | 873 | 873 | 873 | 873 | 873 | 873 | 873 | 873 | 873 |
|  |  |  |  |  |  |  |  |  |  |
| Standard errors in parentheses |  |  |  |  |  |  |  |  |  |
| *** p<0.001, ** p<0.01, * p<0.05, + p<0.1 | | | | | | | | | |

**Supplementary Table S4**. Summary of testing for mediation/suppression effects of the baseline two step reach (TSR) or the ego network using 20 windows beginning in 2002, 2003, and 2004, and ending in 2008, 2009, 2010, 2011, and 2012.

| **Study window** | **Baseline TSR significant?** | **Sign of coefficient** | **Type of effect** |
| --- | --- | --- | --- |
| 2002-2008 | Y | Negative | Suppression |
| 2002-2009 | *N* | *Negative* | *Suppression* |
| 2002-2010 | *N* | *Positive* | *Mediation* |
| 2002-2011 | Y | Positive | Mediation |
| 2002-2012 | Y | Positive | Mediation |
|  |  |  |  |
| 2003-2008 | Y | Negative | Suppression |
| 2003-2009 | Y | Negative | Suppression |
| 2003-2010 | *N* | *Positive* | *Mediation* |
| 2003-2011 | Y | Positive | Mediation |
| 2003-2012 | Y | Positive | Mediation |
|  |  |  |  |
| 2004-2008 | Y | Negative | Suppression |
| 2004-2009 | Y | Positive | Mediation |
| 2004-2010 | Y | Positive | Mediation |
| 2004-2011 | Y | Positive | Mediation |
| 2004-2012 | Y | Positive | Mediation |
|  |  |  |  |
| 2005-2008 | Y | Negative | Suppression |
| 2005-2009 | Y | Negative | Suppression |
| 2005-2010 | Y | Negative | Suppression |
| 2005-2011 | Y | Negative | Suppression |
| 2005-2012 | Y | Negative | Suppression |

Supplementary Figure S1. Frequency of consulting with MICHR in 2006 (zeros or non-consultations not shown).

Supplementary Figure S2. Frequency of consulting with MICHR in 2007 (zeros or non-consultations not shown).

Supplementary Figure S3. Frequency of consulting with MICHR in 2008 (zeros or non-consultations not shown).

Supplementary Figure S4. Frequency of consulting with MICHR in 2009 (zeros or non-consultations not shown).

Supplementary Figure S5. Frequency of consulting with MICHR in 2010 (zeros or non-consultations not shown).
